# The burdens, coping strategies, and unmet needs of family caregivers of Chinese patients with lymphoma receiving commercial CAR-T-cell therapy: A qualitative study

**DOI:** 10.1016/j.apjon.2025.100850

**Published:** 2026-01-06

**Authors:** Xijun Lin, Jiali Liu, Jianwen Chen, Jiamin Tang, Zhicong Huang, Ye Wang, Simei Shi

**Affiliations:** Sun Yat-sen University Cancer Center, State Key Laboratory of Oncology in South China, Collaborative Innovation Center for Cancer Medicine, Guangzhou, China

**Keywords:** Chimeric antigen receptor T cell, Family caregivers, Burden, Coping, Unmet needs, Qualitative research

## Abstract

**Objective:**

This study aimed to describe the caregiving experiences, coping strategies, and unmet health care needs of family caregivers supporting Chinese patients with large B-cell lymphoma receiving commercial chimeric antigen receptor T-cell therapy.

**Methods:**

A qualitative descriptive design was adopted. Semistructured interviews were conducted with 19 primary family caregivers at a university-affiliated cancer centre in southern China. Data were analysed using Braun and Clarke's thematic analysis framework.

**Results:**

Three themes were identified: (1) overwhelming caregiving burden, including financial strain, emotional distress, and role conflict; (2) caregivers' patient-centred coping strategies, including familial financial sacrifice, emotional suppression to protect the patient, and reprioritizing work and personal life for care; and (3) unmet caregiver support needs, including navigating therapy costs without systemic support, gaps in caregiver education and care coordination, and caregivers' emotional support being overlooked in care systems.

**Conclusions:**

Family caregivers of patients receiving this therapy in China experience significant challenges, which are often managed through culturally influenced coping strategies amid limited formal support. A caregiver-inclusive approach (including financial reforms, tailored caregiver education, increased care coordination, and psychosocial services) is essential to support the well-being of family caregivers and optimize treatment outcomes amid evolving oncology trends in China.

## Introduction

Lymphoma, which is classified into Hodgkin lymphoma and non-Hodgkin lymphoma, has imposed a substantial global health burden, with 20.1% of all non-Hodgkin lymphoma patients being observed in China.^1^ Among non-Hodgkin lymphoma subtypes, large B-cell lymphoma remains the most prevalent type, and approximately one-third of large B-cell lymphoma patients subsequently develop relapsed/refractory disease following first-line therapy.^2^ Those patients who are eligible for second-line treatment undergo salvage chemotherapy followed by stem cell transplantation.^3^^,^^4^ Patients who do not respond to salvage therapy or who experience relapse after stem cell transplantation typically die from lymphoma.^3^^,^^5^ Chimeric antigen receptor (CAR)-T-cell therapy has emerged as a revolutionary treatment; specifically, it offers durable remission and markedly extends survival in patients with relapsed/refractory large B-cell lymphoma patients.^5^, ^6^, ^7^ In 2021, two CAR-T-cell products were approved in China, thereby providing new options for Chinese patients with refractory/relapsed large B-cell lymphoma.

Following the extraction, genetic modification, and expansion of patient lymphocytes—a process taking several weeks—patients undergo lymphodepletion before reinfusion.^8^ After infusion, patients are typically hospitalized for approximately three weeks for close monitoring and timely management, as the therapy is associated with a substantial risk of acute toxicity. Two of the most common complications are cytokine release syndrome (CRS) and immune effector cell-associated neurotoxicity syndrome (ICANS), which affect approximately 45% to 50% of patients, with severe conditions (grade ≥ 3) occurring in 10% to 12% of patients.^9^ CRS manifests as a systemic inflammatory response, typically presenting with symptoms such as fever, fatigue, myalgia, hypotension, and hypoxia, and may lead to organ dysfunction.^10^^,^^11^ ICANS is characterized by impaired attention and cerebral oedema.^10^^,^^11^ Furthermore, the costs of CAR-T-cell therapy are substantial: approximately USD 400,000 in the United States and CNY 1,200,000 in China.^12^ In 2024, the national annual per capita disposable income in China was only CNY 41,314,^13^ highlighting the significant financial barrier this therapy poses for most families. In China, commercial CAR-T-cell therapy is accessed mainly in tertiary centres and is predominantly paid for by individuals.^14^ Candidates are identified according to label- and institution-based criteria, with clinicians providing recommendations and risk–benefit and cost information to support shared decision-making by patients and families.^15^ The complexity and high cost of this treatment not only complicate clinical management but also place a substantial burden on family caregivers.^16^^,^^17^

Family caregivers—typically close family members such as spouses, parents, or adult children—play crucial roles in supporting patients receiving CAR-T-cell therapy by assisting with decision-making, monitoring adverse reactions, and managing various nonmedical challenges such as financial barriers.^16^^,^^18^ Caregiver burden, defined as the perceived adverse effect of caregiving on emotional, social, financial, physical, and spiritual functioning, is well documented among cancer caregivers, who often report high levels of anxiety, depression, and sleep disorders.^19^, ^20^, ^21^, ^22^ This burden negatively influences caregivers' health and quality of life, which may indirectly diminish care quality and patient outcomes.^20^^,^^23^, ^24^, ^25^ Quantitative studies have shown that 15% of family caregivers of patients receiving CAR-T-cell therapy experience moderate to severe burdens, and 47.7% exhibit symptoms of depression.^18^^,^^26^^,^^27^

Family caregivers of cancer patients have commonly reported unmet needs regarding health systems and information, patient care and support, leaving them unprepared for caregiving tasks.^28^ This lack of preparedness has not only impeded their ability to manage caregiving challenges but also negatively affected their health.^29^^,^^30^

As more patients opt for CAR-T-cell therapy, understanding the multifaceted challenges that their family caregivers face becomes increasingly important.^31^ However, existing studies on caregivers of those receiving CAR-T-cell therapy remain limited and have predominantly been conducted in Western countries; thus, this experience within the Chinese cultural and health care context remains poorly understood. Accordingly, this qualitative study aimed to explore the experiences of family caregivers of patients receiving commercial CAR-T-cell therapy for lymphoma in China, focusing on their caregiving burdens, coping strategies, and unmet needs, to identify service gaps and inform the development of culturally appropriate and targeted supportive interventions.

## Methods

### Design

This study adopted a qualitative descriptive design. Qualitative descriptions provide comprehensive summaries of phenomena in participants' everyday terms and allow researchers to stay close to the data and to the surface of words and events.^32^ This approach is typically employed to investigate poorly understood phenomena and to generate straightforward, practice-oriented insights that can inform intervention development.^33^ It was therefore chosen for its ability to capture the real-world caregiving burden, coping strategies, and unmet needs of family caregivers within the clinical and cultural context of CAR-T-cell therapy in China. The study was reported in accordance with the Standards for Reporting Qualitative Research (SRQR).^34^

### Ethical considerations

This study was approved by the ethics committee of the study cancer centre (Approval No. B2023-384-01). Written informed consent was obtained from all the participants prior to data collection. If any emotional distress arose during the interview, the interviewer paused the session and offered to take a break or discontinue. A referral pathway to the hospital's psycho-oncology team was in place, although no formal referrals were ultimately needed. To ensure the privacy of the participants and the security of the data, all personal identifiers were removed from the transcripts, and the data were stored in a secure system with restricted access, available only to the research team.

### Setting and participants

The study was conducted between September 2023 and May 2024 at a university-affiliated tertiary cancer centre in southern China. The participants were family caregivers of patients with lymphoma receiving commercial CAR-T-cell therapy. Eligible family caregivers were Mandarin-speaking relatives or partners who had provided unpaid care during the CAR-T-cell treatment period for at least six months and who were aged ≥ 18 years. Family caregivers with diagnosed mental illness or cognitive impairment were excluded. The research team approached family caregivers during patients' hospitalizations for CAR-T-cell infusion to introduce the concept of the study. Those who expressed interest were screened for eligibility, and the study's purpose, procedures, potential risks and benefits, and data confidentiality were clearly explained. To increase diversity, purposive sampling was applied to include caregivers of varying ages, sexes, relationships with the patient, and educational backgrounds. Participation was voluntary and did not affect clinical care, and the participants retained the right to withdraw at any time without penalty. Written informed consent was obtained prior to data collection. A total of 21 eligible family caregivers were approached, two of whom declined due to time constraints at the time of contact. Nineteen family caregivers were enrolled in the study.

### Data collection

Data were collected via semistructured face-to-face interviews conducted in a private room within the hospital, with only the first author and family caregiver present. All the interviews were conducted by the first author—an oncology specialist nurse with a master's degree and extensive CAR-T-cell nursing experience—who has received formal education and training in qualitative research methods. The interviews were scheduled within one month after CAR-T-cell infusion to coincide with patients' follow-up visits to the treatment centre after discharge; moreover, each interview lasted for 35–60 minutes and were audio-recorded with consent. The interview guide (Table 1) was developed on the basis of the study objectives, reviewed by three oncology nursing experts, and revised to incorporate additional follow-up probes such as “*What additional* *support* *would you have wished to have? Why?”* and “*What is the greatest difficulty or challenge you have encountered during your family member's CAR-T-cell therapy?”* The guide was pilot-tested with two family caregivers, after which minor wording adjustments were made for clarity. Each interview was immediately coded, with ongoing review being utilized to determine if new codes were identified. Thematic saturation was defined as the point at which no new codes were identified.^35^ By the 17th interview, no additional codes were identified; moreover, two further interviews (the 18th and 19th interviews) were conducted to verify saturation, after which recruitment was terminated. In addition to the interviews, the participants completed a brief demographic questionnaire, covering sex, age, education, occupation, duration of caregiving, and monthly family income.

**Table 1:** Qualitative interview guide.

● Can you share your understanding of CAR-T-cell therapy? Please describe your experience during the decision-making process for this treatment?
● What difficulties and challenges have you faced during your family's CAR-T-cell therapy? (e.g., emotionally, socially, financially, physically, spiritual functioning)
● What is the greatest difficulty or challenge you have encountered during your family's CAR-T-cell therapy?
● How did you cope with these difficulties or challenges?
● How have these difficulties or challenges affected you?
● Did you receive any support during your family's CAR-T-cell therapy? What kind of support was it? What additional support would you have wished to have? Why?
● How do you perceive CAR-T-cell therapy and its impact on your caregiving experience?

CAR, chimeric antigen receptor.

### Data analysis

All the interviews were transcribed verbatim in Chinese. The transcripts were checked for accuracy, returned to participants for verification, and analysed. Data analysis followed Braun and Clarke's six-phase thematic analysis framework.^36^ Thematic analysis is a systematic method for describing and interpreting qualitative data through code generation and theme development. Owing to its flexibility, thematic analysis can be applied across diverse theoretical and epistemological orientations, research questions and sample sizes and is recognized as a powerful method for understanding experiences.^37^^,^^38^ Themes were actively developed by the research team through iterative coding, comparison, discussion, and refinement. First, the research team familiarized themselves with the transcripts through repeated reading. Next, using NVivo v12, the first author conducted line-by-line open coding to identify significant statements. The second author independently reviewed the coded transcripts to verify coding accuracy and ensure consistency. All the authors then discussed the generated codes and resolved any discrepancies through consensus meetings. Related codes were clustered into descriptive categories, which were further compared and refined into themes. Within each theme, subthemes were identified to capture distinct yet interrelated aspects of caregivers' experiences. These themes and subthemes were reviewed and refined by comparing them against the entire dataset to ensure that each was internally coherent and distinct from the others. Finally, each theme and subtheme was explicitly defined, named, and illustrated through an analytic narrative supported by representative participant extracts. Initial Chinese themes, subthemes, and quotes were independently forwards- and backwards-translated by two professional bilingual translators, and the study team reconciled any discrepancies to finalize the English versions. The research team held regular discussions to reflect on their clinical backgrounds and potential biases, ensuring reflexivity throughout the research process.

### Rigor

Guided by Lincoln and Guba's trustworthiness framework, several strategies were implemented to enhance study rigor.^39^ Credibility was strengthened by employing maximum-variation purposive sampling (age, gender, education) and member checking. The participants received their verbatim transcripts along with summaries of preliminary themes and were invited to verify their accuracy. All the researchers were oncology specialist nurses. The first author, who holds a master's degree and has extensive CAR-T-cell training experience, conducted all the interviews and performed the initial inductive and semantic coding. The second author, who holds a PhD and has expertise in qualitative research methods, independently reviewed the transcripts and codes. Coding disagreements were resolved through regular consensus discussions among the research team. Finally, detailed descriptions of the study context, caregiving environment, and participant characteristics facilitated readers' judgements regarding the transferability of findings to other settings.

## Results

### Demographic characteristics

A total of 19 participants, including 13 females and 6 males with an average age of 46 years, were recruited, including the children, spouses, and parents of lymphoma patients. The sociodemographic information of the participants and patients is presented in Table 2.

**Table 2:** Sociodemographic characteristics of the caregivers and patients.

Caregivers								Patients			
ID	Sex	Age (years)	Education	Occupation	Time being a caregiver (years)	Relationship to patient	Monthly family income (CNY)	Patient has commercial insurance	Patient's age (years)	Severity of adverse events^a^	ADL score^b^
C1	Female	56	Bachelor	Civil servant	2	Wife	< 5000	No	51	Grade 2 CRS	90
C2	Female	46	Bachelor	Teacher	7	Wife	< 5000	No	47	Grade 1 CRS	95
C3	Female	52	Bachelor	Doctor	3	Wife	5001–10,000	No	52	Grade 1 CRS	95
C4	Female	50	Senior high school	Retailer	2	Mother	< 5000	No	25	Grade 2 CRS	65
C5	Male	48	Bachelor	Clerk	9	Husband	> 15,000	No	47	Grade 1 CRS	95
C6	Female	50	Junior high school	Trader	3	Wife	5001–10,000	No	53	Grade 1 CRS	100
C7	Male	54	Senior high school	Trader	6	Husband	10,001–15,000	No	54	Grade 1 CRS	95
C8	Male	67	Junior high school	Retired	1.5	Husband	< 5000	No	66	Grade 1 CRS	45
C9	Female	34	Bachelor	Civil servant	1.5	Daughter	5001–10,000	No	68	Grade 2 CRS	95
C10	Female	37	Bachelor	Teacher	7 months	Wife	< 5000	Yes	38	Grade 2 CRS	55
C11	Female	47	Bachelor	Clerk	1	Wife	< 5000	No	47	Grade 4 CRS/Grade 4 ICANS	20
C12	Female	35	Bachelor	Clerk	1	Wife	< 5000	No	39	Grade 1 CRS	100
C13	Male	40	Bachelor	Clerk	4	Son	> 15,000	No	72	Grade 1 CRS	95
C14	Male	54	Senior high school	Trader	1.5	Father	10,001–15,000	No	30	Grade 1 CRS	95
C15	Female	26	Bachelor	Unemployed	1	Wife	10,001–15,000	Yes	38	Grade 1 CRS/Grade 1 ICANS	100
C16	Male	37	Junior high school	Retailer	1.5	Son	5001–10,000	No	55	Grade 1 CRS	95
C17	Female	70	Senior high school	Retired	1	Wife	< 5000	No	78	Grade 2 CRS	95
C18	Female	57	Junior high school	Trader	1	Wife	10,001–15,000	No	60	Grade 1 CRS	100
C19	Female	20	Bachelor	Student	1	Daughter	< 5000	Yes	50	Grade 1 CRS	100

CAR, chimeric antigen receptor.

a CRS, cytokine release syndrome; ICANS, immune effector cell-associated neurotoxicity syndrome.

b ADL, Activities of Daily Living (0–100); higher scores indicate better functional status.

### Themes identified

A total of three themes and nine subthemes were identified, capturing the experiences of family caregivers of patients receiving commercial CAR-T-cell therapy in the Chinese cultural context. These included (1) overwhelming caregiving burden, (2) caregivers' patient-centred coping strategies, and (3) unmet caregiver support needs. A detailed summary of the themes and subthemes is presented in Table 3.

**Table 3:** Themes, subthemes and example codes.

Themes	Subthemes	Example codes
Overwhelming caregiving burden	1. Financial strain across the treatment journey	High cost of CAR-T-cell therapy; accumulated financial burden from previous treatments; loss of income due to long-term caregiving and patient unemployment; travel, lodging, and daily living expenses during treatment; commercial insurance reducing burden.
	2. Emotional distress	Fear of severe CAR-T-related complications; anxiety about treatment effectiveness; delaying CAR-T-cell therapy due to fear of risks; struggling to decide when the disease is worsening; regret for not choosing CAR-T-cell therapy earlier; anxiety about future medical expenses.
	3. Role conflicts	Frequent work absence for hospital care; fear of job loss due to prolonged absence; shifting childcare or eldercare duties to extended family members; guilt over children's declining school performance.
Caregivers' patient-centred coping strategies	1. Familial financial sacrifice to enable treatment	Selling property to pay for CAR-T; depleting lifetime savings; borrowing money from relatives or friends; reducing children's extracurricular expenses; cancelling vacations to save money; cutting nonessential household spending; adopting a frugal lifestyle.
	2. Emotional suppression to protect the patient	Hiding personal anxiety and distress from the patient; feigning optimism despite bad news; withholding worries from ageing parents; rarely confiding in friends or others; belief that expressing emotions is unnecessary or inappropriate.
	3. Reprioritizing work and personal life for care	Taking unpaid leave to provide full-time care; switching to flexible or low demand jobs; declining help from extended family; belief that caregiving must be provided by immediate family.
Unmet caregiver support needs	1. Navigating CAR-T-Cell therapy costs without systemic support	No national insurance coverage for CAR-T-cell therapy; absence of regional insurance reimbursement; desire for government financial support; restrictions on using existing personal funds; need for staged payment or subsidies.
	2. Gaps in caregiver education and care coordination	Insufficient information on procedures and risks; limited communication with clinicians; no designated point-of-contact for urgent issues; no support when complications occurred at home; lack of knowledge regarding who to direct questions to after discharge; need for a dedicated coordinator/nurse navigator; community clinics not being connected to tertiary hospitals.
	3. Caregivers' emotional support being overlooked in care systems	Emotional needs rarely being acknowledged by health care staff; prioritizing patient's emotions over own-being; self-silencing to avoid burdening others; uncertainty about where to seek mental health support; lack of caregiver-specific counselling services or hotlines.

CAR, chimeric antigen receptor.

#### Theme 1: overwhelming caregiving burden

1 Financial strain across the treatment journey

Most of the family caregivers described financial strain as their most immediate and overwhelming stressor. Some noted that the cost of CAR-T-cell therapy far exceeded the financial capacity of an average Chinese household. For most families, the economic burden did not begin with CAR-T-cell therapy; it was compounded by substantial costs accumulated across previous treatment stages, including medications, genetic testing, imaging, and complication management. By the time CAR-T-cell therapy was considered, families were already under significant financial strain.

“CAR-T-cell therapy was too expensive. Even with my high income, I felt immense financial pressure. Many people in my hometown couldn't earn 1.2 million yuan in their lifetime.” (C14)

“After going through this ordeal, my father had no money left. My parents worked hard and saved their entire lives, and now all the money has been spent on treatment.” (C9)

Participants also described additional financial strain due to loss of income, as patients were unable to continue working after becoming ill. Furthermore, travelling long distances to tertiary hospitals offering CAR-T-cell therapy led to substantial transportation and accommodation costs.

“For the past two years, we've spent most of our time in the hospital, so I couldn't focus on my business. I haven't gained any new clients or earned money.” (C6)

“Daily expenses such as travel, hotels, and food were daunting. Most people couldn't afford it.” (C16)

While financial pressure was common across households, it was particularly pronounced among lower-income families and attenuated when supplementary commercial insurance was available. In our sample, three patients had supplementary commercial insurance that covered most treatment costs, including CAR-T-cell therapy. Family caregivers of these insured patients reported reduced financial burden.

“Fortunately, my father purchased commercial insurance a few years ago, which covered all the CAR-T-cell treatment costs. Without it, we could never have afforded the therapy. The expenses are reimbursable, so the pressure isn't as great.” (C19)

2 Emotional distress

Family caregivers experienced profound emotional strain, partly due to fears of severe treatment-related complications and uncertainty about treatment efficacy. Prior to initiating CAR-T-cell therapy, many family caregivers actively sought information about possible adverse reactions and the likelihood of treatment success. This process often heightened their anxiety, as they frequently encountered accounts of serious and sometimes fatal side effects, as well as reports of poor therapeutic outcomes. Even family caregivers with medical backgrounds expressed similar concerns:

“As a physician myself, I worry most about CRS. Although my own hospital offers CAR-T, I didn't dare have the treatment there. This hospital is larger, and I believe they have more experience managing complications.” (C3)

Such fears contributed to prolonged hesitation. Some delayed treatment despite medical recommendations, uncertain whether the patient could withstand the risks. As the disease progressed and CAR-T-cell therapy emerged as a potentially more promising choice among limited alternatives, they ultimately decided to proceed with the therapy.

“The doctor had recommended CAR-T therapy for my mother six months ago, but we hesitated for a long time after hearing that some people had died from it. We feared it might hasten her decline.” (C16)

Family caregivers reported intense feelings of guilt and helplessness as the patient's condition deteriorated. Some participants expressed regret about not choosing CAR-T-cell therapy earlier, believing that the delay may have led to missed treatment opportunities. This sense of self-blame further intensified their psychological burden.

“My son's health is deteriorating. He can barely walk to the bathroom. Witnessing his suffering breaks my heart. …. We should have chosen CAR-T earlier. I deeply regret not coming sooner to find a doctor who could offer CAR-T therapy.” (Crying, C4)

The economic burden was not exclusively a monetary strain; it also resulted in a heavy psychological burden. Some caregivers felt anxious about the high costs of CAR-T-cell therapy and worried about their ability to afford future medical expenses and living costs.

“I feel very anxious. We've almost run out of our savings, and I don't know how we'll cover the medical bills ahead. We still have two children to care for, and the future feels completely uncertain.” (C12)

3 Role conflicts

Family caregivers described occupying the role of the family's “middle generation"—responsible both for ageing parents and for raising children—while also maintaining employment. They reported constant struggles to balance work, intensive caregiving for CAR-T-cell therapy recipients, and ongoing responsibilities for both children and older family members. Many participants recounted the difficulty of keeping their jobs while providing round-the-clock care. Frequent hospital visits and prolonged admissions often meant repeated leave requests, reduced work performance, and increased anxiety about job security.

“Every time he is hospitalized, I need several days off. I've used up all my annual leave and taken over a month of personal leave. If I take more time off, I might be fired.” (Crying, C3)

Family caregivers also described the influence on their wider family roles. The demands of intensive caregiving often left them with little time or energy to care for their children or ageing parents. As a result, some individuals had to shift family responsibilities to other relatives, which frequently triggered feelings of guilt and emotional conflict.

“I haven't been able to care for my two children since my husband fell ill. The teacher called saying their academic performance had declined. Hearing about their poor school performance makes me cry; I feel deeply sorry.” (C10)

Overall, this theme highlights the cumulative and multifaceted burden experienced by family caregivers of CAR-T-cell therapy recipients. The intensive demands of CAR-T-cell therapy placed them under intertwined pressures of financial hardship, emotional strain, and conflicting family and occupational responsibilities.

#### Theme 2: caregivers' patient-centred coping strategies

##### *1 Familial financial sacrifice to enable treatment*

To make CAR-T-cell therapy feasible considering its substantial out-of-pocket costs, many families described taking active steps to reallocate household resources, including selling property, depleting savings, borrowing money, and reducing discretionary spending.

“To provide him with treatment, we sold our house. Several of our grandchildren used to attend many extracurricular classes, which was another major expense, but they've now stopped …. The treatment is expensive, but we are a family, and we will do our best.” (C17)

In addition to these larger financial shifts, caregivers reported modifying daily consumption habits to accommodate ongoing treatment costs. These adjustments included cancelling vacations, limiting nonessential purchases, and adopting more frugal lifestyles.

“In the past, we used to travel during holidays, but now we have cut unnecessary expenses and saved money for her medical treatment.” (C8)

Such decisions were described as necessary and unquestioned. These choices were not weighed or negotiated but were considered necessary for families to do in a situation of life-threatening illness.

“He is the continuation of our lives. The cost of over one million yuan is significant, but nothing is more important than his life. We will do our best to save him.” (C14)

Together, these accounts reflect how caregivers restructured their financial lives in both large and small ways, driven not only by practical necessity but also by a deeply held sense of familial obligation. Despite the financial strain, families demonstrated collective resolve and a clear willingness to prioritize the patient's survival above all else.

2 Emotional suppression to protect the patient

Nearly all the participants described suppressing their worries and anxieties in front of the patient. Regardless of how distressed they felt, family caregivers made a conscious effort to remain calm. Even when they received unfavourable news, caregivers deliberately maintained an appearance of optimism in front of the patient to protect the patient from further emotional distress. This self-restraint was consistently described as a way of protecting the patient's psychological well-being:

“I often suppress my anxiety because expressing it would only make my son worry more. In front of him, I always appear optimistic.” (C4)

In addition to concealing their emotions from the patient, most family caregivers refrained from discussing their struggles with others. Some explained that their families rarely talked openly about feelings, while others worried that sharing would only burden others. A few participants mentioned occasionally confiding in close friends, but such moments were rare. For many, emotional expression was seen as unnecessary or even inappropriate.

“My parents are getting old, so I don't tell them anything—I don't want them to worry. No one can understand what we're going through. Talking about it doesn't really help.” (C5)

This ongoing self-silencing helped maintain emotional stability for patients but resulted in family caregivers experiencing prolonged helplessness and emotional isolation.

3 Reprioritizing work and personal life for care

Despite the ongoing demands of work and family, many family caregivers described caregiving as their unquestioned top priority. To ensure a continuous presence during hospitalization and recovery, they proactively adjusted their professional and personal lives—taking extended or unpaid leave, switching to flexible positions, or even suspending employment altogether. These choices were often made at significant personal and economic cost but were rarely viewed with resentment.

“To care for him, I took a temporary job that would not take much time. My focus is now his care, with work taking a backseat.” (C15)

In addition to workplace adjustments, some family caregivers declined assistance from other relatives, insisting on personally providing care. This strong preference for hands-on involvement stemmed from an internalized sense of duty, shaped by the belief that caregiving should remain within the immediate family and ideally be performed by the closest kin. For these family caregivers, family care was not a task to be delegated but a personal commitment rooted in moral and cultural expectations.

“Although my husband's siblings offered help, I felt it was my responsibility. Unless I became ill myself, I believed it was up to me to provide his care.” (C10)

In summary, prioritizing caregiving above all else was not just a pragmatic choice for Chinese families but also a behavioural norm deeply embedded in the ethics and collective identity of family life, reflecting both a sense of duty and the willingness to sacrifice individual interests for loved ones.

#### Theme 3: unmet caregiver support needs

1 Navigating CAR-T-cell therapy costs without systemic support

Family caregivers expressed frustration over the lack of institutional support to offset the high cost of CAR-T-cell therapy. Nearly all the participants mentioned a desire for governmental assistance, particularly through the inclusion of CAR-T-cell therapy in national or regional insurance schemes. Some family caregivers suggested that insurance coverage would reduce treatment delays and increase access for families with limited income.

“We have health insurance and purchased Hui Min Bao (a regional insurance plan), but none of it covers CAR-T. We hope the government can shoulder part of the costs for patients with critical illnesses—otherwise, very few people can afford CAR-T therapy.” (C11)

In addition to insurance coverage, participants highlighted the need for more flexible financing pathways. They suggested that staged payments, low-interest medical loans, or access to existing personal resources—such as a housing provident fund (a compulsory housing savings account jointly contributed by employers and employees in China)—could help alleviate financial burdens.

“Actually, our combined housing provident fund is enough to cover CAR-T. My wife could have received CAR-T therapy at least six months earlier if it could be used for that. But under current rules, we can only withdraw it to pay for housing expenses, not for medical care. That's absurd. Isn't life more important than a house?” (C7)

2 Gaps in caregiver education and care coordination

Family caregivers commonly reported that busy clinicians had limited time for in-depth communication, resulting in insufficient and delayed information about CAR-T-cell therapy, especially regarding treatment procedures, potential adverse effects, and postdischarge care. Although some participants sought answers online, most expressed greater trust in professional guidance from their physicians. This lack of structured education from the health care team left many of the caregivers feeling unprepared and anxious about managing the needs of the patient at home.

“I thought CAR-T therapy would start right after the decision was made, but I didn't expect such a long preparation process. I only realized the complexity and duration after we began. If I had known earlier, we would have chosen this treatment much sooner." (C11)

In addition to the caregiver education gap, family caregivers reported difficulty accessing timely support from the clinical team when urgent questions or complications arose. The absence of a designated point of contact—particularly during the cell manufacturing period and after hospital discharge—left many families to navigate potential emergencies alone. Several participants emphasized the need for a dedicated care coordinator or liaison to offer timely updates, facilitate communication, and provide reassurance during these critical and uncertain phases of the treatment process.

“We were waiting at home for the CAR-T-cells to be manufactured, but her disease was progressing rapidly. The National Day holiday was coming, and even if we went to the hospital, we couldn't find her doctor. If something like sudden bleeding happened again, like last time, I wouldn't know what to do. I really need a professional person to stay in touch. It's reassuring when medical staff are there for us." (C13)

After discharge, this sense of isolation persisted. Despite the presence of community health centres in many neighbourhoods, family caregivers reported that these facilities had little connection to the tertiary hospitals where CAR-T-cell therapy was performed, and meaningful follow-up was not provided. With no formal handovers or communication systems in place, families were left to navigate complex posttreatment care independently.

“After we went back home, no one from the community hospital contacted us. It's very hard for us to rely on them for help.” (C10)

3 Caregivers' emotional support overlooked in care systems

Despite the high emotional demands of caregiving, few participants received any formal psychological support. Family caregivers consistently reported that health care professionals focused almost exclusively on patients, with little attention given to family members' emotional well-being. Routine psychological screening or counselling referrals for family caregivers were virtually nonexistent.

“No one ever asked me how I was doing. It was all about the treatment, the tests, the patient—but never about us.” (C1)

Even when family caregivers felt emotionally overwhelmed, most did not actively seek professional support. Some participants felt that their own psychological needs were secondary to those of the patient and therefore not deserving of attention.

“Sometimes I get so anxious that I cannot sleep, but it is not a big problem. The most important thing is now to take good care of my husband.” (C18)

Other participants were unsure where to access help or questioned whether such services were even intended for family caregivers. Owing to the lack of dedicated resources, such as counselling hotlines, peer support groups, or caregiver-focused mental health services, many participants were left to manage their distress in isolation.

“The psychological pressure is definitely there. Besides worrying about whether my wife can recover from CAR-T-cell therapy, my mother also has lung cancer. I'm constantly afraid that one day I might lose them both. I don't even know who I can talk to—there's no one who can really help me.” (C5)

This theme highlights caregivers' unmet needs in multiple domains. They lacked governmental financial assistance for CAR-T-cell therapy, received limited professional guidance during complex treatment phases, and rarely acknowledged their own psychological well-being. The absence of accessible and continuous support left many of these individuals feeling isolated and unprepared to manage the demanding caregiving process.

## Discussion

### Main findings

This study offers qualitative insights into the real-world experiences of family caregivers of Chinese patients with lymphoma receiving commercial CAR-T-cell therapy, thereby contributing evidence from an underrepresented context. Three central features were identified: considerable financial, emotional, and role-related strain; patient-centred coping; and unmet needs in terms of financial protection, information and care continuity, and psychological support. Overall, these findings highlight the substantial demands placed on families throughout the CAR-T-cell treatment trajectory.

Nearly all caregivers who were required to pay for CAR-T-cell therapy out-of-pocket identified the financial burden as their primary challenge. Unlike recent reports from the United Kingdom, where caregivers of CAR-T-cell therapy recipients rarely mentioned financial concerns.^17^ This contrast likely reflects differences in health care financing structures. In China, commercial CAR-T-cell therapy is not yet covered by the national basic medical insurance system; thus, families pay treatment costs out of pocket. These direct expenses are compounded by income loss and additional costs for travel, lodging, and prolonged caregiving. In contrast, in some Western and Asian countries, public reimbursement mechanisms that cover most CAR-T-cell therapy expenses have been implemented, thereby reducing families' financial vulnerability.^40^, ^41^, ^42^ In China, however, only high-income families can realistically afford access to such life-saving innovations, while younger or middle-income households often face untenable choices, such as selling assets, incurring significant debt, or forgoing treatment. Chinese caregivers remain more vulnerable to catastrophic health spending and treatment-related impoverishment.^43^

In addition to financial strain, many family caregivers described increased anxiety and emotional exhaustion. These experiences seemed to be influenced by the substantial financial burden, and this factor has been noted in previous studies that have reported greater financial toxicity being associated with increased anxiety and psychological distress.^44^, ^45^, ^46^ Concerns about CAR-T-cell-related toxicity and uncertainty regarding treatment outcomes also contributed to this emotional burden, particularly as CAR-T-cell therapy was often perceived as a last resort after conventional treatments had failed. Similar findings have been reported among caregivers of CAR-T-cell therapy recipients in Western countries, who also reported emotional distress.^17^^,^^18^^,^^26^ In our study, the fear of economic consequences and severe side effects sometimes led to hesitation or delays in decision-making. As caregivers played a central role throughout the treatment trajectory—from gathering information and consulting physicians to deciding whether to pursue CAR-T-cell therapy—such hesitation often resulted in self-blame when complications or disease progression occurred. Even when outcomes were beyond their control, many internalized guilt and responsibility, a phenomenon echoed in the global caregiving literature.^47^^,^^48^ In the Chinese sociocultural context, this emotional burden may be further intensified by expectations that place family members at the centre of medical decision-making.^49^^,^^50^ Rooted in values of filial piety and moral accountability, caregivers often viewed treatment choices as moral duties rather than shared responsibilities, which amplified their distress and self-reproach.^51^

Role conflict posed an additional and often underrecognized source of family caregiver stress. Many participants were part of the “sandwich generation” simultaneously caring for ageing parents, supporting children, and maintaining employment, as previously reported in the literature.^52^ The intensive demands of CAR-T-cell care—often requiring full-time, hands-on involvement—exacerbated this tension. Without access to formal caregiving services or institutional support, these multiple roles were shouldered alone, heightening the risk of burnout and occupational strain.^53^^,^^54^ This reflects not only the individual burden of caregiving but also deeper structural gaps in China's long-term care infrastructure and social support systems.

Family caregivers adopted various patient-centred coping strategies, such as making financial sacrifices, suppressing their emotions, and restructuring work and family roles, to manage the demands of CAR-T-cell therapy. These strategies often reflected deeply rooted Confucian values, particularly filial piety and familial obligation, framing caregiving as a moral responsibility rather than merely a personal burden.^55^, ^56^, ^57^ Financially, most families chose to draw on available resources for treatment, such as selling assets, borrowing money, or adopting a more frugal lifestyle, which is consistent with previous research.^58^ Although these sacrifices were perceived as meaningful demonstrations of love and familial commitment, they also risked household financial instability, anxiety about accumulated debt, and vulnerability to future crises.^59^^,^^60^ This suggests that while familial sacrifice may foster solidarity, targeted financial support interventions are necessary to mitigate its long-term adverse consequences.

At the emotional level, family caregivers commonly suppressed their own worries and anxieties in front of the patient. Although emotional suppression has been noted in other cultures, it is particularly prominent in China, where emotional restraint and family harmony are culturally valued.^61^ In contrast, the Western literature emphasizes the benefits of open communication within families and between patients and health care providers for emotional well-being and informed decision-making.^62^^,^^63^ While culturally appropriate, the practice of emotional concealment among Chinese family caregivers may lead to feelings of isolation and prolonged emotional strain.

When facing conflicts among work, family, and caregiving responsibilities, family caregivers in our study often reduced or even suspended their employment to prioritize patient care. Despite the competing demands, most participants preferred to engage in direct, hands-on caregiving, rarely delegating tasks even when outside support was available. This reluctance reflected both strong family loyalty—the belief that care should be provided by the closest family members—and the lack of established formal caregiving support systems in China. In contrast, Western families may utilize respite care or other primary care support, which helps distribute the caregiving burden and alleviate stress.^64^^,^^65^

Although these coping strategies demonstrated family caregivers' resilience and strong familial commitment, they often come at substantial personal costs that may erode family caregivers' health, autonomy, and long-term caregiving capacity.^60^^,^^66^ Thus, structural interventions integrating financial protection and psychosocial support are urgently needed to sustain family caregivers' roles while preserving their well-being.

Nearly all the participants expressed an urgent need for governmental intervention, particularly through the inclusion of CAR-T-cell therapy in national or regional medical insurance reimbursement schemes and the development of flexible financing mechanisms beyond traditional insurance, such as staged payments and low-interest medical loans. However, with China's rapidly ageing population, the sustainability of its national basic medical insurance fund is becoming increasingly challenging.^67^ To increase access to high-cost yet clinically effective treatments such as CAR-T-cell therapy, policymakers should establish a multitiered health care financing system in China, including expanding basic insurance to partially reimburse eligible patients, negotiating price reductions with CAR-T-cell manufacturers or adopting value-based pricing linked to outcomes, expanding the coverage and payout levels of commercial health insurance, and enhancing public awareness of supplemental insurance as a valuable complement to public health care coverage.^68^

Moreover, many family caregivers reported uncertainty due to limited consultation time and inadequate information about CAR-T-cell therapy procedures, complications, and home care. These results support previous findings that information provision and family caregiver training are among the most frequently reported unmet needs in cancer care.^69^^,^^70^ Considering the complexity and novelty of CAR-T-cell therapy, structured family caregiver education programs should be developed. These programs should be tailored to China's health care context, standardized in content, staged according to the treatment timeline, and supplemented by ongoing professional guidance.^16^^,^^71^ Family caregivers also lacked consistent contact with clinical teams during critical nonhospitalization periods, such as the cell manufacturing phase and postdischarge monitoring for CRS or ICANS. China's high-volume, hospital-centred health care system—often marked by fragmented care pathways and limited integration with community services—poses challenges for ensuring continuity of care for family caregivers.^72^^,^^73^ To bridge these gaps, continuity of care should be strengthened through dedicated CAR-T-cell therapy nurse navigators, accessible 24-hour helplines, and formalized referral pathways to trained community providers.

The findings indicated that clinical care typically focuses on patient needs and overlooks family caregivers' emotional well-being. However, systematic reviews and meta-analyses have indicated that structured psychosocial interventions, such as counselling, psychotherapy, and stress management, could effectively reduce family caregiver burden, alleviate depression, and improve quality of life.^74^, ^75^, ^76^ Health care providers and policymakers should therefore formally recognize family caregivers as integral to CAR-T-cell therapy, integrating routine psychosocial screening into clinical workflows from diagnosis onwards to identify at-risk family caregivers and provide timely support.^29^ Systems such as clear referral pathways, caregiver-specific support groups, and free psychosocial counselling should be established to reduce emotional distress and strengthen family caregivers' resilience amidst the intensive demands of CAR-T-cell therapy.^77^

Collectively, addressing these unmet needs requires comprehensive structural interventions and coordinated policy efforts, targeting financial accessibility, clinical communication, and emotional support systems, to sustain family caregiver well-being and optimize patient outcomes.

### Strengths and limitations

This study has several strengths. It provides rich qualitative data from family caregivers of patients receiving commercial CAR-T-cell therapy in China, which is currently an underexplored context. The findings reveal culturally shaped caregiving burdens and coping strategies and offer contextually grounded insights into gaps within China's oncology and social health care systems. Finally, the systematic and rigorous thematic analysis ensured analytical transparency and credibility. However, several limitations must be noted. Participants were recruited from a single tertiary hospital in China, which may limit the transferability of the findings to other regions or care settings. As a qualitative study, the sample size was relatively small and may not reflect the full diversity of family caregiver experiences, particularly across different socioeconomic or rural–urban backgrounds. Most participants in this study were family caregivers of patients who had the financial means to access CAR-T-cell therapy. Those unable to afford the treatment—and therefore potentially facing even greater distress or barriers—were not included, which may lead to an underestimation of the broader challenges faced by socioeconomically disadvantaged families. Finally, the family caregivers' economic backgrounds likely shaped how the financial burden was perceived; this context should be considered when interpreting transferability. To address these limitations, in future research, multicentre designs could be adopted, hospitals in rural settings and lower-income families unable to access CAR-T-cell therapy could be included, and longitudinal studies on family caregiver adaptation over time could be conducted.

## Conclusions

This study revealed that family caregivers of patients receiving CAR-T-cell therapy in China face intense, multifaceted challenges—financially, emotionally, and socially—while relying heavily on culturally shaped coping strategies and receiving limited formal support. As CAR-T-cell therapy continues to evolve from experimental to mainstream, addressing the needs of these family caregivers is critical. A caregiver-inclusive approach—incorporating financial reform, structured education, coordinated communication, and psychosocial care—is essential for sustaining both family caregiver well-being and ensuring successful treatment in China's rapidly transforming oncology landscape.

## CRediT authorship contribution statement

**Xijun Lin:** Conceptualization, Formal Analysis, Investigation, Data curation, Writing – Original Draft, Writing – Review & Editing. **Jiali Liu:** Formal Analysis, Data Curation, Writing – Review & Editing. **Jianwen Chen:** Data Curation, Writing – Review & Editing. **Jiamin Tang:** Data Curation. **Zhicong Huang:** Data Curation. **Ye Wang:** Data Curation, Supervision. **Simei Shi:** Project Administration. All authors have read and approved the final manuscript.

## Ethics statement

The study was approved by the Ethics Committee of Sun Yat-sen University Cancer Center (Approval No. B2023-384-01) and was conducted in accordance with the 1964 Helsinki Declaration and its later amendments or comparable ethical standards. All participants provided written informed consent.

## Data availability statement

The data that support the findings of this study are available on request from the corresponding author. The data are not publicly available due to privacy concerns of participants.

## Declaration of generative AI and AI-assisted technologies in the writing process

No AI tools/services were used during the preparation of this work.

## Funding

This study was supported by the Nurses Association of Guangdong Province (Grant No. gdshsxh2023qn04) and the Natural Science Foundation of Guangzhou (Grant No. 2023A04J1775). The funders had no role in considering the study design or in the collection, analysis, interpretation of data, writing of the report, or decision to submit the article for publication.

## Declaration of competing interest

The authors declare no conflict of interest.
